# Overlapping Mechanisms of Peripheral Nerve Regeneration and Angiogenesis Following Sciatic Nerve Transection

**DOI:** 10.3389/fncel.2017.00323

**Published:** 2017-10-11

**Authors:** Hongkui Wang, Hui Zhu, Qi Guo, Tianmei Qian, Ping Zhang, Shiying Li, Chengbin Xue, Xiaosong Gu

**Affiliations:** ^1^Key Laboratory of Neuroregeneration of Jiangsu and Ministry of Education, Co-innovation Center of Neuroregeneration, Nantong University, Nantong, China; ^2^Jiangsu Clinical Medicine Center of Tissue Engineering and Nerve Injury Repair, Research Center of Clinical Medicine, Affiliated Hospital of Nantong University, Nantong, China

**Keywords:** peripheral nerve regeneration, angiogenesis, bioinformatic analysis, Ingenuity Pathway Analysis (IPA), molecular mechanism, sciatic nerve transection

## Abstract

Peripheral nervous system owns the ability of self-regeneration, mainly in its regenerative microenvironment including vascular network reconstruction. More recently, more attentions have been given to the close relationship between tissue regeneration and angiogenesis. To explore the overlap of molecular mechanisms and key regulation molecules between peripheral nerve regeneration and angiogenesis post peripheral nerve injury, integrative and bioinformatic analysis was carried out for microarray data of proximal stumps after sciatic nerve transection in SD rats. Nerve regeneration and angiogenesis were activated at 1 day immediately after sciatic nerve transection simultaneously. The more obvious changes of transcription regulators and canonical pathways suggested a phase transition between 1 and 4 days of both nerve regeneration and angiogenesis after sciatic nerve transection. Furthermore, 16 differentially expressed genes participated in significant biological processes of both nerve regeneration and angiogenesis, a few of which were validated by qPCR and immunofluorescent staining. It was demonstrated that STAT3, EPHB3, and Cdc42 co-expressed in Schwann cells and vascular endothelial cells to play a key role in regulation of nerve regeneration and angiogenesis simultaneously response to sciatic nerve transection. We provide a framework for understanding biological processes and precise molecular correlations between peripheral nerve regeneration and angiogenesis after peripheral nerve transection. Our work serves as an experimental basis and a valuable resource to further understand molecular mechanisms that define nerve injury-induced micro-environmental variation for achieving desired peripheral nerve regeneration.

## Introduction

Peripheral nervous system (PNS) owns its ability to autonomously regenerate compared with central nervous system (CNS) with a limited regenerative potential for a few factors inhibiting nerve regeneration (Webber and Zochodne, 2010; Egawa et al., 2017). Peripheral nerve regeneration has many influencing factors including the type and extent of injury, the time and manner of injury repair and also the patient’s comprehensive situation and so on. So far, autologous nerve repair is still a gold standard for peripheral nerve injury (Stenberg et al., 2016), although more understanding of mechanisms of peripheral nerve regeneration help us to provide effective treatments for patients with peripheral nerve injury (Gu et al., 2014; Hu et al., 2016; Lindholm et al., 2017). People realize that there are other key factors not resolved, in which lack of blood supply is one of important constraint and bottleneck factors (Jain et al., 2005; Novosel et al., 2011), especially for long-distance peripheral nerve defects. More recently, it is reported that vascular endothelial cells guide the regeneration of peripheral nerve axons directly confirming the direct relationship between nerve regeneration and angiogenesis (Cattin et al., 2015). Through the blood vessel three-dimensional reconstructions, we indeed observed the lack of angiogenesis in the middle of tissue-engineered nerves and the difference of maturation of vascular network between tissue-engineered nerves and autologous nerves repairing sciatic nerve defects in rats (Wang et al., 2016).

So, a more efficient growth-permissive microenvironment especially enough blood supply should be constructed to achieve the desired effect of peripheral nerve regeneration by the control of effective and adverse conditions on the basis of a deep understanding of molecular mechanisms during peripheral nerve regeneration. Herein, our hypothesis is that the modulation of angiogenesis could be a key strategy for achieving better axonal regeneration post peripheral nerve injury. Intervention of the key molecules regulating both peripheral nerve regeneration and angiogenesis can be more effective to achieve the desired peripheral nerve regeneration effect. Mechanisms of molecular regulation of angiogenesis in the process of development and tumorigenesis have many studies (Song et al., 2017; Wild et al., 2017). Numerous molecules influencing and regulating angiogenesis were reported (Carmeliet and Jain, 2011; Potente et al., 2011). However, the basic researches on angiogenesis during regeneration of tissues and organs are still relatively small. The specific molecular regulations of angiogenesis following peripheral nerve injury have remained unknown, for instance, relationship of critical biological processes, interactions and collaboration of key molecules, common important regulators, and interconnections of signaling pathways.

Regenerated axons sprout from the proximal nerve stump and extend to the distal stump post peripheral nerve injury. Cell body of neuron continues to synthesize new proteins and other substances transporting to axons to provide a material basis for axonal regeneration. The proximal nerve stump contains more regenerative informations than the distal nerve stump after peripheral nerve injury. To figure out the overlap of molecular mechanisms and key regulation molecules, our experiment focuses on the analysis and presentation of molecule correlations between peripheral nerve regeneration and angiogenesis post sciatic nerve transection. We carried out integrative approaches to analyze the microarray data of proximal nerve stumps, including gene expression profiling combined with multi-level bioinformatic analysis and also experimental validations. We compared the performance of differentially expressed genes and built protein–protein interaction (PPI) networks for peripheral nerve regeneration and angiogenesis. Furthermore, the key regulation molecules were validated by qPCR and immunofluorescent staining. Our work provide a framework for deep understanding the precise molecular networks and pathways, as well as an experimental basis for choice of key regulation molecules, involved in both peripheral axonal regeneration and angiogenesis after transecting the sciatic nerve.

## Materials and Methods

### Animal and Surgery

Adult male Sprague-Dawley (SD) rats (200–220 g, provided by the Experimental Animal Center of Nantong University) were divided into 5 groups of 15 rats each randomly. The animals were deeply anesthetized by an intraperitoneal injection of a compound anesthetic (chloral hydrate 4.25 g, magnesium sulfate 2.12 g, sodium pentobarbital 886 mg, ethanol 14.25 mL, and propylene glycol 33.8 mL in 100 mL) at a dose of 0.2–0.3 mL/100 g. The sciatic nerve was identified and exposed through an incision on the lateral aspect of the mid-thigh of left hind limb. Then, the sciatic nerve was transected in the central position of femur followed by its retraction of two stumps without any repair. The animals recovered in warm cages after the surgery. All experimental protocols were approved by the Administration Committee of Experimental Animals, Jiangsu Province, China, in accordance with the US National Institute of Health (NIH) Guide for the Care and Use of Laboratory Animals published by the US National Academy of Sciences.

### Sample Preparation and Microarray

The nerve samples were harvested according to the previous methods with some modifications (Li et al., 2013). Briefly, the proximal sciatic nerve stumps about 5 mm were collected at 1, 4, 7, and 14 days after the nerve transection surgery and normal sciatic nerve segments in the corresponding position, respectively. Total RNA was extracted using Trizol (Life technologies, Carlsbad, CA, United States) according to the manufacturer’s instructions. RNA quality of each tissue sample was determined using Agilent Bioanalyzer 2100 (Agilent technologies, Santa Clara, CA, United States) and Nanodrop ND1000 spectrophotometer (NanoDrop Technologies, Wilmington, DE, United States). Microarray analysis was conducted by an Agilent Microarray Scanner (Agilent Technologies) and the subsequent data compiled with Agilent feature extraction software. All steps from RNA amplification to the final scanner output were performed by National Engineering Center for Biochip at Shanghai (China). Three biological replicates were designed and performed for each time point to reduce experimental errors. Array normalizations and error detection were conducted using Silicon Genetics’ GeneSpring GX Version 10.0 (Agilent Technologies) via the enhanced Agilent feature extraction import preprocessor. All data is MIAME compliant and the raw data has been deposited in a MIAME compliant database (NCBI Accession number: Series GSE30165), as detailed on the website^1^.

### Bioinformatic Analysis

The differential gene expression profile at different time points post nerve transection was provided in **Supplementary Data S1**. Only 10% of probes that showed highest variation across analyzed populations were collected for bioinformatic analysis (Mingueneau et al., 2013). Z-score (standard score) and hierarchical clustering were calculated on log2-transformed mean-centered datasets filtered for expression values greater than 128 in any subsets. Hierarchical clustering was performed with the multi-experiment viewer (MeV 4.9^2^). Venn diagrams were performed with Venn-Diagrams online tool^3^. The molecular network, function prediction, transcription regulator, and pathway analysis of the expression data were conducted with QIAGEN’s Ingenuity Pathway Analysis (IPA, QIAGEN Redwood City^4^) (molecular network, function prediction, and pathway analysis: genes with FC ± 2.0; transcription regulator: genes with FC ± 1.5).

### Real-time RT-PCR

Total RNA was extracted. Reverse-transcribed complementary DNA was synthesized with the Prime-Script RT Reagent Kit (TaKaRa, Dalian, China). qPCR was performed with SYBR Premix Ex Taq (TaKaRa, Dalian, China). The relative expression level of the selected gene was calculated using the comparative 2^-ΔΔCt^ method. The sequences of primer pairs are as follows: *Stat3* (5′-3′) forward, GTCTCAGAACCTTGTGTCG; reverse, TGGGAACCAAGCACATAGAAT. *Csf1r* (5′-3′) forward, TGATGTGTGGTCCTACGG; reverse, ATCCATCCTTCACCAGTTTG. *Ephb3* (5′-3′) forward, AGACTGACTCAGAGAGCC; reverse, ACAACACACGCATACATACC. *Myh10* (5′-3′) forward, AGTTCTGTGTTCATGTGGC; reverse, CCGAGACAGCGATCACTA. *Ephb2* (5′-3′) forward, GCTTTAACACAGTGGATGAGT; reverse, CCATCATCATCTGAGATACGAC. *Cdh2* (5′-3′) forward, ATTTGGACTTTGGATTCAGGT; reverse, ATTCTAACTACAGCTCAACGG. *Gapdh* (5′-3′) forward, GCGAGATCCCGCTAACATCA; reverse, CTCGTGGTTCACACCCATCA. The data obtained from three independent experiments.

### Immunofluorescence Validation

The tissue samples about 5 mm of proximal sciatic nerve stumps at 1, 4, 7, and 14 days after surgery, also the normal sciatic nerves were harvested for longitudinal sections. The immunofluorescent triple-staining was performed using rabbit anti-STAT3 antibody (1:50 dilution, Proteintech), rabbit anti-EPHB3 antibody (1:100 dilution, Abcam), rabbit anti-Cdc42 antibody (1:100 dilution, Abcam), mouse anti-NF200 antibody (1:200 dilution, Sigma), mouse anti-S100 antibody (1:500 dilution, Sigma), mouse anti-RECA1 antibody (1:25 dilution, Abcam), and Hoechst 33342 (1:5000 dilution, Life Technologies), respectively. The nerve sections were incubated with the primary antibody at 4°C for 24 h, followed by further reaction with the secondary antibody (Goat anti-Mouse IgG-Alex-488, 1:500; Goat anti-Mouse IgG-Cy3, 1:100; Goat anti-Rabbit IgG-Cy3, 1: 100) at room temperature for 1 h. At last, the nerve sections were observed and photographed under a fluorescence microscopy (AxioImager M2, Zeiss).

### Statistical Analysis

For statistical analysis, the data were replicated in at least three repeat experiments. The data are presented as means ± SEM. Multiple comparisons were carried out with one-way ANOVA plus Scheffe’s *post hoc t*-test using the Stata 7.0 software package (Stata Corp., College Station, TX, United States). Differences were considered significant at ^∗^*p*-value < 0.05, ^∗∗^*p*-value < 0.01, and ^∗∗∗^*p*-value < 0.001.

## Results

### Trends of Biological Processes during Nerve Regeneration and Angiogenesis Post Sciatic Nerve Transection

**FIGURE 1:**
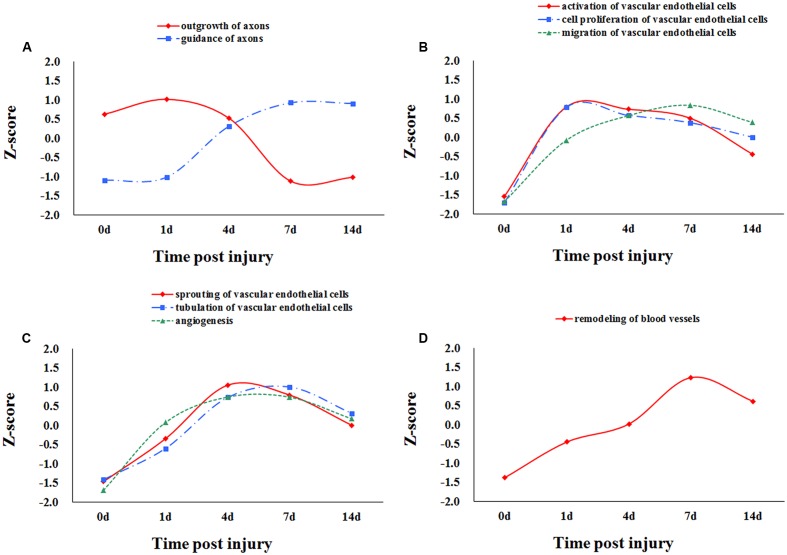
Dynamic changes of biological processes of nerve regeneration and angiogenesis post sciatic nerve transection. The Z-scores calculated using the average expression profiles of differentially expressed genes represent the dynamic trends of major biological processes of nerve regeneration and angiogenesis at different time points post nerve transection. Outgrowth of axons was activated at early stages followed by guidance of axons later **(A)**. All biological processes of angiogenesis were activated at 1 day with different primary activation periods. Vascular endothelial cells and cell proliferation of vascular endothelial cells were mainly at early stages, and migration of vascular endothelial cells was relatively late **(B)**. Activation of angiogenesis, sprouting of vascular endothelial cells, and tubulation of vascular endothelial cells were mainly at middle stages **(C)**. Remodeling of blood vessels was mainly at late stages **(D)**.

The significant biological processes of nerve regeneration and angiogenesis were selected to present the overall changes in these two interested aspects at different time points post sciatic nerve transection. Z-scores calculated with the average expression profiles of differentially expressed genes (three duplicate data corresponding to three duplicate samples at different time points) involved in different biological processes reflect the trends of different functions. The Z-score curves showed the trends of peripheral nerve regeneration: outgrowth of axons at early stages followed by guidance of axons a litter later (**Figure 1A**). The major biological processes of angiogenesis were divided into three groups according to time sequence of angiogenesis. All biological processes of angiogenesis were activated at 1 day with different primary activation periods. Activation of vascular endothelial cells and cell proliferation of vascular endothelial cells were mainly at early stages, and the major process of migration of vascular endothelial cells was relatively late (**Figure 1B**). Activation of angiogenesis, sprouting of vascular endothelial cells, and tubulation of vascular endothelial cells were mainly at middle stages (**Figure 1C**). Remodeling of blood vessels was activated mainly at late stages (**Figure 1D**). Both nerve regeneration and angiogenesis were activated at 1 day immediately after sciatic nerve transection indicating the close correlation.

### Molecule Correlations between Nerve Regeneration and Angiogenesis Post Sciatic Nerve Transection

**FIGURE 2:**
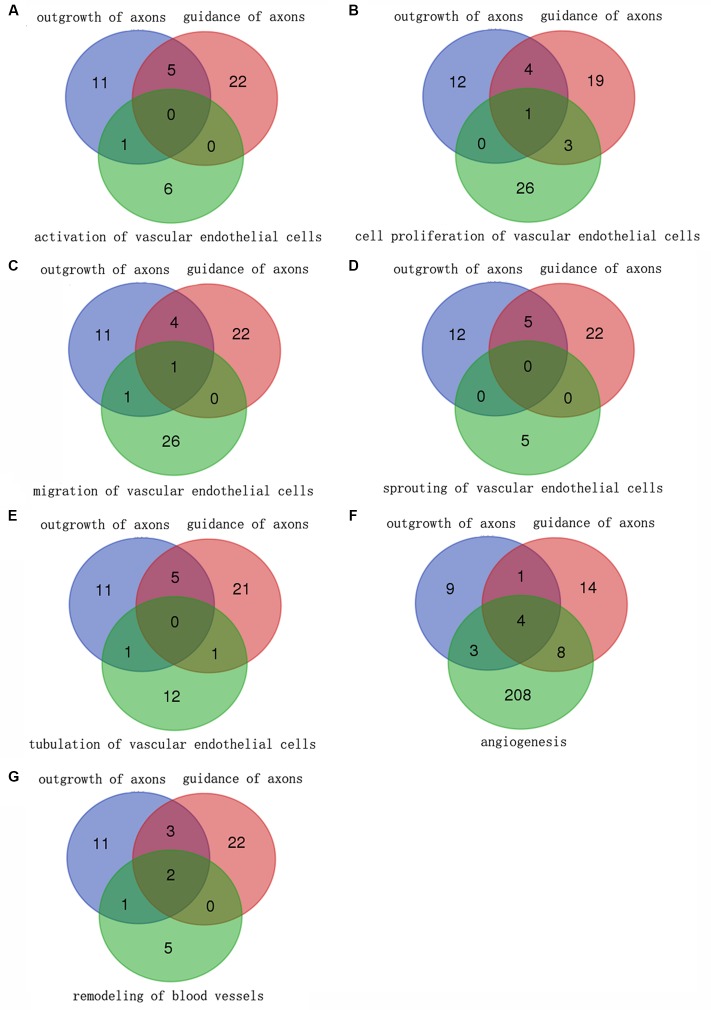
Correlations of differentially expressed genes between nerve regeneration and angiogenesis. The Venn diagrams showed comparisons of the number of differentially expressed genes involved in different biological processes in different colors between nerve regeneration and angiogenesis. Molecules in different biological processes of angiogenesis (green) were intersected with molecules in outgrowth of axons (blue) and guidance of axons (red) **(A–G)**. Molecules participating in biological processes were displayed in numbers. The figure in overlap of different color circles indicated the number of molecules involved in all these biological processes. A few different molecules participated in nerve regeneration and also the biological process of angiogenesis except sprouting of vascular endothelial cells. Altogether 16 genes were involved in both nerve regeneration and angiogenesis post sciatic nerve transection.

To figure out the molecules regulating nerve regeneration meanwhile controlling angiogenesis, which are more critical relative to the molecules regulating just a single biological process, the Venn diagrams were performed to analysis the molecule correlations (the same and different molecules) between outgrowth of axons and guidance of axons during nerve regeneration and the significant biological process during angiogenesis, respectively (**Figure 2**). Molecules in different biological processes of angiogenesis were intersected with molecules in outgrowth of axons and guidance of axons. Several different molecules participated in nerve regeneration and also the biological process of angiogenesis except sprouting of vascular endothelial cells. Altogether 16 genes were involved in both nerve regeneration and angiogenesis post sciatic nerve transection by the sum of common molecules in every panel (1 + 4 + 2 + 0 + 2 + 15 + 3 = 27) followed by removal of the duplicate items (Rac1^∗^2 + *Runx3^∗^*2 + *Stat3^∗^*2 + *Ngfb^∗^*1 + *Ptk2^∗^*1 + *Ephb3^∗^*1 + *Ephb2^∗^*1 + *Nrp2^∗^*1 = 11).

### Analysis of Genes Involved in Both Nerve Regeneration and Angiogenesis Post Sciatic Nerve Transection

**FIGURE 3:**
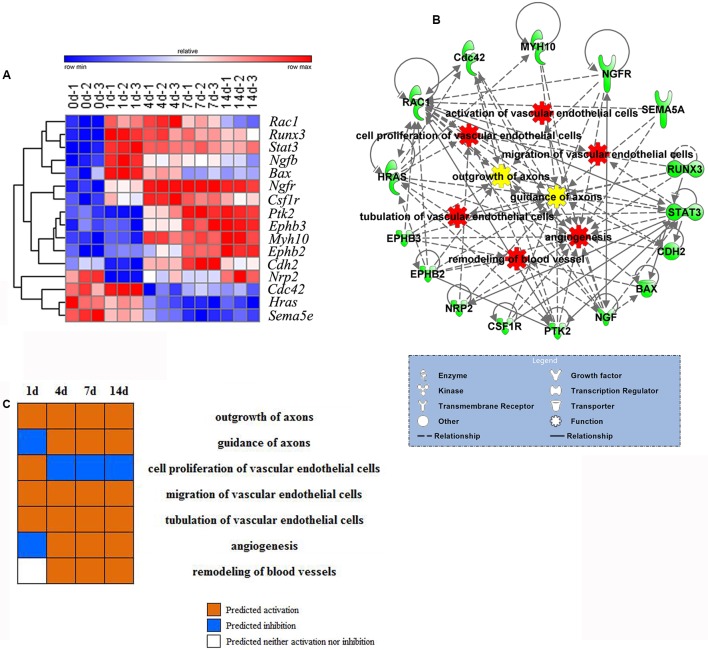
Analysis of differentially expressed genes involved in both nerve regeneration and angiogenesis. **(A)** Heatmap of dynamic gene expressions for both nerve regeneration and angiogenesis. Red represented varying degrees of up-regulation. Blue represented varying degrees of down-regulation. **(B)** Protein–protein interaction (PPI) network for differentially expressed genes of nerve regeneration and angiogenesis including correlations between genes and biological processes. Molecules (green) connected with each other by direct (solid line) or indirect (dotted line) relationships, and participated in biological processes of both nerve regeneration (yellow) and angiogenesis (red). **(C)** Functional predictions (with 16 differentially expressed genes) of the biological processes of nerve regeneration and angiogenesis at different time points post nerve transection. The nerve regeneration and angiogenesis were activated at 1 day immediately after sciatic nerve transection simultaneously. Orange represented predicted activation. Blue represented predicted inhibition. White represented predicted neither activation nor inhibition.

The 16 differentially expressed genes in both nerve regeneration and angiogenesis were selected for further analysis. The dynamic expression levels of these 16 genes including two transcription regulators *Runx3* and *Stat3* were showed in the clustered heatmap with three different patterns: up-regulation in the early, in the middle, and in the late (**Figure 3A**). To investigate the relationships between these proteins encoded by these 16 genes and also relationships between these proteins and functions, PPI network was constructed by IPA, which also clearly revealed their molecular types including enzyme, growth factor, kinase, transcription regulator, transmembrane receptor, transporter, and other types of molecules (**Figure 3B**). PPI network demonstrated that the selected molecules point to both biological processes of nerve regeneration and angiogenesis, and connect with each other by direct or indirect relationships. Gene expression changes including up-regulation and down-regulation affect biological functions ultimately. The major biological processes of nerve regeneration and angiogenesis were predicted with 16 genes by IPA to demonstrate the changes of activation and inhibition at different time points post sciatic nerve transection (**Figure 3C**) (no prediction outcomes of activation of vascular endothelial cells due to too little genes involved). The prediction by IPA also demonstrated that the nerve regeneration and angiogenesis were activated at 1 day immediately after sciatic nerve transection simultaneously.

### Cascade Regulations of Transcription Regulators of Nerve Regeneration and Angiogenesis Post Sciatic Nerve Transection

**FIGURE 4:**
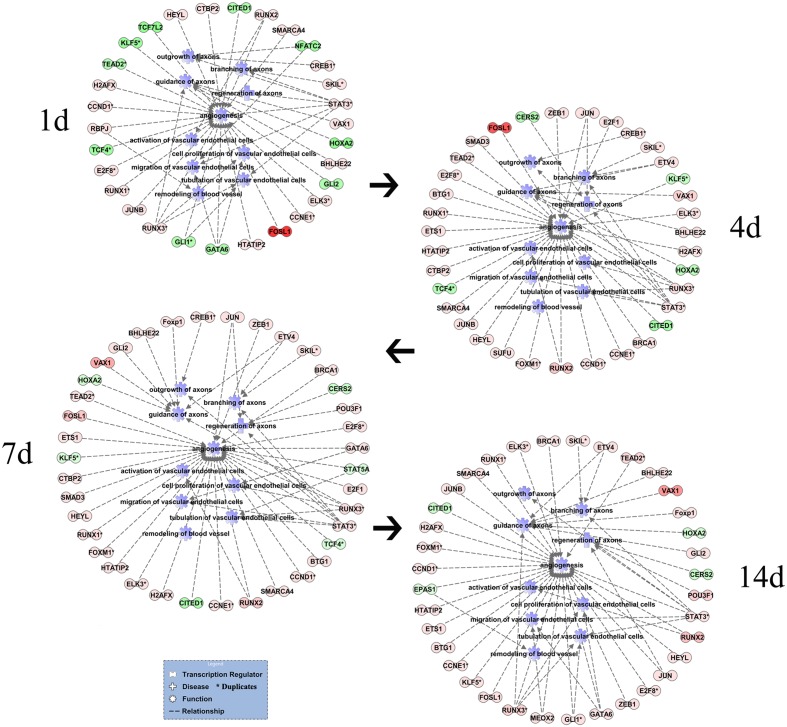
Cascade network of differentially expressed transcription regulators of nerve regeneration and angiogenesis post sciatic nerve transection. The interaction networks for differentially expressed transcription regulators (FC ± 1.5) of nerve regeneration and angiogenesis at different time points post nerve transection. In addition to RUNX3 and STAT3 (FC ± 2.0), there are also NFATC2 and JUN regulating both nerve regeneration and angiogenesis with smaller expression changes. The number of transcription regulators had a sustained increase at 4 and 7 days, however, a slightly reduction at 14 days post sciatic nerve transection. The transcription factors were mainly up-regulated at each time point except more down-regulated transcription factors at 1 day post surgery. Red represented varying degrees of up-regulation. Green represented varying degrees of down-regulation. Gene identifiers marked with an asterisk indicated that multiple identifiers in the dataset file map to a single gene in the global molecular network.

Transcription regulators, commonly initiating lots of biological processes or signaling pathways in early phase, play important roles in regulation of gene expressions. The transcription regulators (FC ± 1.5) regulating nerve regeneration and angiogenesis were connected with functions to demonstrate the cascade changes of transcription regulation at different time points post sciatic nerve transection (**Figure 4**). In addition to RUNX3 and STAT3 (FC ± 2.0), there are also NFATC2 and JUN (FC ± 1.5) regulating both nerve regeneration and angiogenesis with smaller expression changes. NFATC2 regulated outgrowth of axons and migration of vascular endothelial cells with the down-regulation expression at 1 day. JUN regulated regeneration of axons and angiogenesis with the up-regulation expressions at 4, 7, and 14 days. The numbers of transcription regulators were 30, 35, 38, and 36 at 1, 4, 7, and 14 days, respectively. The number of transcription regulators had a sustained increase at 4 and 7 days, however, a slightly reduction at 14 days post sciatic nerve transection. The transcription factors were mainly up-regulated at each time point except more 10 down-regulated transcription factors at 1 day post surgery. The more obvious dynamic changes of transcription factors including the number and expression suggested that there was a phase transition of nerve regeneration and angiogenesis between 1 and 4 days after nerve transection.

### Canonical Pathways Enrichment Analysis of Nervous and Cardiovascular System Post Sciatic Nerve Transection

**FIGURE 5:**
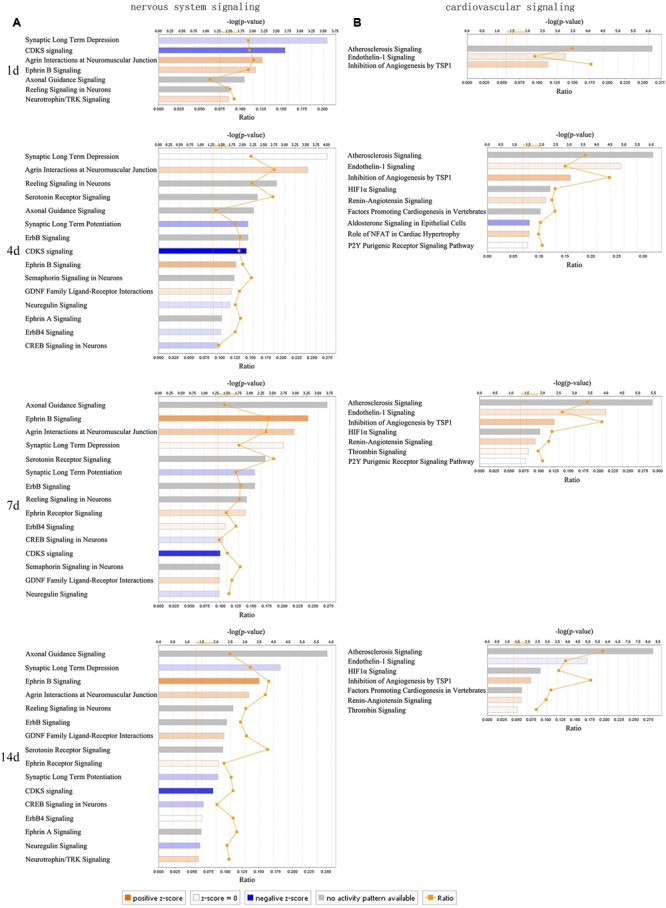
Dynamic changes of canonical pathways of nervous and cardiovascular system post sciatic nerve transection. **(A)** The changes of major canonical pathways of nervous system signaling at different time points post nerve transection. **(B)** The changes of major canonical pathways of cardiovascular signaling at different time points post nerve transection. Agrin interactions at neuromuscular junction (NMJ) and Ephrin B signaling were successively activated (z-score ≥ 2), while synaptic long term depression and CDK5 signaling were successively inhibited (z-score ≤ –2) **(A)**. Inhibition of angiogenesis by TSP1 was successively activated (z-score ≥ 2). Endothelin-1 signaling was activated (z-score ≥ 2) at 1, 4, and 7 days, but inhibited at 14 days **(B)**. Each bar represented a pathway with significance of enrichment determined using –log (p) value. Orange, a positive z-score ≥ 2, represented the activation of canonical pathway. Blue, a negative z-score ≤ –2, represented the inhibition of canonical pathway.

Molecules interact with each other including the upstream and downstream in signaling pathways to influence biological functions. Canonical pathways enrichment analysis was carried out by IPA at a pathway level of coordinate expression changes rather than focusing on a single gene. The number of canonical pathways exceeding the threshold of nervous system had a big increase at 4 days post nerve transection. Agrin interactions at neuromuscular junction (NMJ) and Ephrin B signaling were successively activated (z-score ≥ 2), while synaptic long term depression and CDK5 signaling were successively inhibited (z-score ≤-2) post surgery (**Figure 5A**). As a signaling factor deposited at the nerve stump, agrin could be transported to the nerve endings with axon regeneration to increase the formation of NMJ. The number of canonical pathways exceeding the threshold of cardiovascular system had a slight increase at 4 days, followed by a slight decrease at 7 and 14 days post nerve transection. Inhibition of angiogenesis by TSP1 was successively activated (z-score ≥ 2). Endothelin-1 signaling was activated (z-score ≥ 2) at 1, 4, and 7 days, but inhibited at 14 days post surgery (**Figure 5B**).

### qPCR and Histochemical Validation of Selected Genes of Both Nerve Regeneration and Angiogenesis Post Sciatic Nerve Transection

**FIGURE 6:**
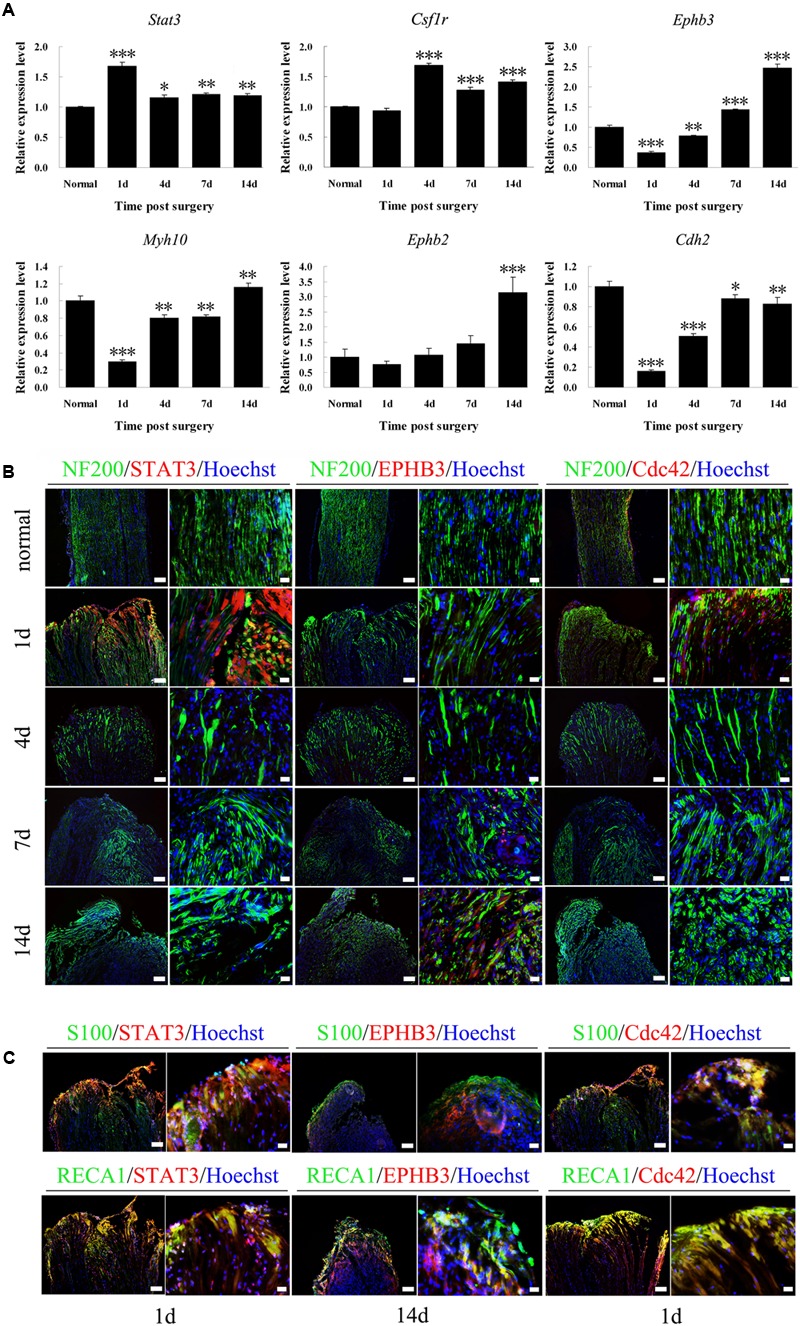
qPCR and histochemical validations of selected genes post sciatic nerve transection. **(A)** Histograms showed the qPCR of relative mRNA expressions of *Stat3*, *Csf1r*, *Ephb3*, *Myh10*, *Ephb2,* and *Cdh2*, which were normalized to *Gapdh*. ^∗^*p* < 0.05, ^∗∗^*p* < 0.01, and ^∗∗∗^*p* < 0.0001 versus the normal control. **(B)** Immunofluorescent staining of anti-STAT3, anti-EPHB3, and anti-Cdc42 (red) merged with anti-NF-200 (green) and Hoechst 33342 (blue) at 1, 4, 7, and 14 days post nerve transection and normal sciatic nerve of the longitudinal sections, respectively. STAT3 was expressed in a small amount in axons. Scale bar, 100 μm (low magnification) and 20 μm (high magnification). **(C)** Immunofluorescent staining of anti-STAT3, anti-EPHB3, and anti-Cdc42 (red) merged with anti-S100 and anti-RECA1 (green), respectively. STAT3 was expressed mainly in Schwann cells and vascular endothelial cells. EPHB3 was expressed mainly in vascular endothelial cells, however, had a small expression in Schwann cells. Cdc42 was expressed in both Schwann cells and vascular endothelial cells. Scale bar, 100 μm (low magnification) and 20 μm (high magnification).

Several genes were selected for qPCR validation including *Stat3*, *Csf1r*, *Ephb3*, *Myh10*, *Ephb2,* and *Cdh2* (^∗^*p* < 0.05, ^∗∗^*p* < 0.01, and ^∗∗∗^*p* < 0.0001 versus the normal control) (**Figure 6A**). The data are demonstrated as means ± SEM (**Supplementary Data S2**). The expression levels of genes selected had different trends of changes post sciatic nerve transection. *Stat3* increased after surgery immediately followed a decrease gradually. *Csf1r* increased from 4 to 14 days after surgery. *Cdh2* decreased followed by an increase and slightly declined again after surgery. *Ephb3*, *Myh10,* and *Ephb2* had another trend of changes with an increase gradually following a decrease after surgery. To validate the expression and tissue localization of proteins encoded by genes, the immunofluorescent triple-staining was performed for STAT3, EPHB3, and Cdc42 with the greater differential expressions and different trends of changes. The regenerated axons extended from the proximal nerve stump to distal nerve stump gradually over time (**Supplementary Figure S1**). STAT3 and Cdc42 showed visible positive signals of expressions in tissue and cell level at 1 day post nerve transection. EPHB3 showed visible positive signals of expressions in tissue and cell level at 7 days and especially at 14 days post nerve transection (**Figure 6B**). STAT3 was expressed in a small amount in axons (NF200) (**Figure 6B**), and expressed mainly in Schwann cells (S100) and vascular endothelial cells (RECA1) (**Figure 6C**). EPHB3 was expressed mainly in vascular endothelial cells, however, had a small expression in Schwann cells (**Figure 6C**). Cdc42 was expressed in both Schwann cells and vascular endothelial cells (**Figure 6C**).

## Discussion

Peripheral nerve regeneration (PNS) maintains its ability to regenerate, which consists of a series of complicated cellular events and molecular pathways, driven by differential expressions of a tremendous number of genes post peripheral nerve injury (Abe and Cavalli, 2008). Blood vessel is an important support system for human bodies. Rapid and adequate vascular network reconstruction is a prerequisite and guarantee for tissue regeneration and physiological function restoration (Makarevich et al., 2015; Rouwkema and Khademhosseini, 2016; Li et al., 2017). After peripheral nerve injury, axons of proximal stump regenerate and distal stump experience Wallerian degeneration because of loss of cell body nutrition. We previously profiled global mRNA expression changes, and demonstrated the dynamic changes of important biological processes and time-dependent expressions of key genes of proximal stump post sciatic nerve transection (Li et al., 2013). Here we further focused on the close correlations of molecular regulations between peripheral nerve regeneration and angiogenesis to provide a precise understanding of molecular mechanism of peripheral nerve regeneration and to achieve better effects of peripheral nerve regeneration.

**FIGURE 7:**
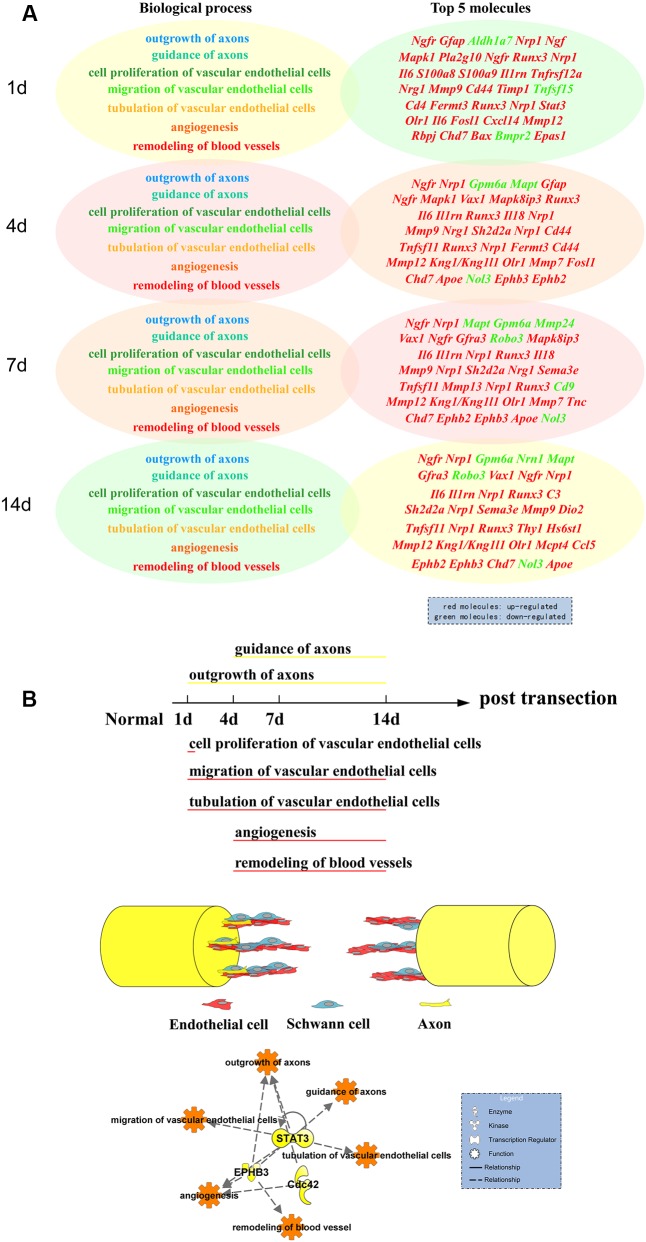
Schematic diagram of functional and molecular changes of nerve regeneration and angiogenesis post sciatic nerve transection. **(A)** Dynamic changes of top 5 differentially expressed molecules of different biological processes of nerve regeneration and angiogenesis post nerve transection. Up-regulated genes primarily played important roles in angiogenesis; however, both up-regulated and down-regulated genes (*Aldh1a7, Gpm6a, Mapt, Mmp24, Nrn1, and Robo3*) played key roles in nerve regeneration. Red represented molecules up-regulated. Green represented molecules down-regulated. **(B)** The phases of major biological processes of nerve regeneration and angiogenesis following the changes of different time points post sciatic nerve transection regulated by the key molecules STAT3, EPHB3, and Cdc42: outgrowth of axons (1–14 days), guidance of axons (4–14 days), cell proliferation of vascular endothelial cells (1 day), migration of vascular endothelial cells (1–14 days), tubulation of vascular endothelial cells (1–14 days), angiogenesis (4–14 days), and remodeling of blood vessels (4–14 days). Finally the basic mode of peripheral nerve regeneration: blood vessel endothelial cells guide Schwann cells for axon regeneration after sciatic nerve transection.

The dynamic trends of the main biological processes of peripheral nerve regeneration and angiogenesis displayed by the Z-score showed their mutual cooperation during whole process of nerve regeneration. By the analysis of Venn diagrams, there were 16 differentially expressed genes involved in both peripheral nerve regeneration and angiogenesis. These key molecules include enzymes (*Rac1* and *Cdc42*) regulating the growth of cell pseudopodia (Potente et al., 2011; Yamao et al., 2015; Schulz et al., 2016) and guidance signals (*Ephb3*, *Ephb2*, and *Sema5a*) (Carmeliet and Jain, 2011; Park and Lee, 2015). The relationships between selected differentially expressed genes and also biological processes were demonstrated by the PPI network. There were direct or indirect interactions between 16 molecules regulating nerve regeneration and angiogenesis. The trends of nerve regeneration and angiogenesis predicted with selected genes by IPA, which could exclude the influence of irrelevant genes, were consistent with those calculated by Z-scores. The predictions by IPA also demonstrated simultaneous activations of peripheral nerve regeneration and angiogenesis at early stages response to nerve injury. Furthermore, the dynamic display of transcription regulators and canonical pathways of peripheral nerve regeneration and angiogenesis provides a new and more comprehensive perspective to understand molecular mechanisms of peripheral nerve regeneration. Many important transcription factors also play a key regulatory role in our sciatic nerve transection models, including c-Jun (JUN), STAT3, RUNX2, and RUNX3. c-Jun participates in biological processes of angiogenesis and axon regeneration. The recent research shows that c-Jun is central to the reprogramming of myelin and non-myelin (Remak) Schwann cells to repair cells after injury (Jessen and Mirsky, 2016). The results indicate that c-Jun is also one of the most important regulators of post-traumatic nerve regeneration. RUNX3 regulates the migration and invasion of vascular endothelial cells, and controls the axonal projection (Inoue et al., 2002; Chen et al., 2013). After nerve transection, the up-regulation of agrin in proximal stump activated the pathway of agrin interactions at NMJ. Agrin, released by motor neurons, triggers and increases the formation of NMJ by stimulating MuSK, a receptor tyrosine kinase expressed in skeletal muscle (Hallock et al., 2010; Tezuka et al., 2014). Endothelin-1, produced by vascular endothelial cells, increases proliferation also migration of cultured HUVEC cells and stimulates neovascularization (Salani et al., 2000), meanwhile regulates proliferation and contraction of vascular smooth muscle cells (Ljuca and Drevensek, 2010; Ou et al., 2014). In order to verify whether the key genes were translated into proteins to play their biological roles, the immunofluorescent staining was performed at the tissue cell level. STAT3, EPHB3, and Cdc42 representing different expression patterns were validated for their important roles in peripheral nerve regeneration and angiogenesis. STAT3, as a transcription regulator, plays a regulatory role in many biological processes involving axon regeneration, microvascular endothelial cell migration, and tube formation (Yahata et al., 2003; Bareyre et al., 2011). It is reported that EPHB3 is necessary for axon guidance, axonal plasticity and regrowth, blood vessel sprouting, and blood vessel remodeling (Cowan et al., 2000; Daniel and Abrahamson, 2000; Liu et al., 2006); and Cdc42 plays an important role in axon outgrowth, proliferation and migration of Schwann cells, and migration of endothelial cells (Yamauchi et al., 2003; Qian et al., 2004; Govek et al., 2005; Benninger et al., 2007). For the first time, the results demonstrated STAT3, EPHB3, and Cdc42 co-expressed mainly in Schwann cells and vascular endothelial cells, suggesting their participations in both peripheral nerve regeneration and angiogenesis post sciatic nerve transection. Finally, schematic diagram demonstrated the top 5 molecules changes (**Figure 7A**), phase changes and key molecular regulations (**Figure 7B**) of significant biological processes of peripheral nerve regeneration and angiogenesis after sciatic nerve transection. In general, mainly up-regulated genes played important roles in angiogenesis; however, both up-regulated and down-regulated genes played key roles in nerve regeneration. Our hypothesis is that blood vessel endothelial cells guide Schwann cells for axon regeneration (**Figure 7B**) after sciatic nerve transection under an important regulation of STAT3, EPHB3, and Cdc42 (Cattin et al., 2015).

Our data describe the injury-induced local microenvironment molecular changes including transcription regulators and canonical pathways, and especially reveal the key molecules (STAT3, EPHB3, and Cdc42) regulating both peripheral nerve regeneration and angiogenesis in proximal nerve stump. The accurate understanding of molecular mechanisms is the prerequisite and basis for regulations of peripheral nerve regeneration. A large number of molecules cooperate with each other to regulate the regeneration process following peripheral nerve injury in an orderly manner. We will not be able to improve the healing effect of peripheral nerve injury feasibly and purposely in clinic, if focus on the entire extremely large and complex molecular regulation networks. The molecules regulating multiple biological functions are more critical and important than the molecules regulating just a single biological process, so that the artificial regulations of multifunctional molecules for angiogenesis and nerve regeneration are more feasible and targeted. The key molecules including transcription regulators regulating both angiogenesis and neural regeneration can further improve the quality of peripheral nerve repair in clinic by promoting the reconstruction of vascular networks, which could be equal to and even surpass the effect of autologous nerve repair. Our work could serve as a valuable basis and a novel perspective to reveal molecular mechanisms of peripheral nerve regeneration for the effective regulations of regenerative microenvironment.

## Author Contributions

XG and CX conceived and designed the experiments. HW, QG, PZ, and HZ performed the experiments. HW and TQ analyzed the data. SL and CX contributed reagents/materials/analysis tools. HW and CX wrote and revised the manuscript. All authors have read and approved the final manuscript.

## Conflict of Interest Statement

The authors declare that the research was conducted in the absence of any commercial or financial relationships that could be construed as a potential conflict of interest.

## Footnotes

1 http://www.ncbi.nlm.nih.gov/geo/query/acc.cgi?acc=GSE30165

2 http://www.tm4.org/

3 http://bioinformatics.psb.ugent.be/software

4 www.qiagen.com/ingenuity

## Supplementary Material

FIGURE S1 Axonal regeneration of proximal nerve stump post sciatic nerve transection. Immunofluorescent staining of anti-NF-200 (red) at 1, 4, 7, and 14 days post nerve transection of longitudinal sections, respectively. The regenerated axons extended to the distal stump gradually over time. The forefront of regenerated axons growing from proximal nerve stump could be seen obviously at 7 and 14 days after nerve transection. Scale bar, 75 μm.

DATA S1 The differential gene expression profile following sciatic nerve transection.

DATA S2 The data of qPCR validation.

## References

[B1] Abe N. Cavalli V. (2008). Nerve injury signaling. *Curr. Opin. Neurobiol.* 18 276–283. 10.1016/j.conb.2008.06.005 18655834 PMC2633416

[B2] Bareyre F. M. Garzorz N. Lang C. Misgeld T. Büning H. Kerschensteiner M. (2011). In vivo imaging reveals a phase-specific role of STAT3 during central and peripheral nervous system axon regeneration. *Proc. Natl. Acad. Sci. U.S.A.* 108 6282–6287. 10.1073/pnas.1015239108 21447717 PMC3076857

[B3] Benninger Y. Thurnherr T. Pereira J. A. Krause S. Wu X. Chrostek-Grashoff A. (2007). Essential and distinct roles for cdc42 and rac1 in the regulation of Schwann cell biology during peripheral nervous system development. *J. Cell Biol.* 177 1051–1061. 10.1083/jcb.200610108 17576798 PMC2064365

[B4] Carmeliet P. Jain R. K. (2011). Molecular mechanisms and clinical applications of angiogenesis. *Nature* 473 298–307. 10.1038/nature10144 21593862 PMC4049445

[B5] Cattin A. L. Burden J. J. Van Emmenis L. Mackenzie F. E. Hoving J. J. Garcia Calavia N. (2015). Macrophage-induced blood vessels guide Schwann cell-mediated regeneration of peripheral nerves. *Cell* 162 1127–1139. 10.1016/j.cell.2015.07.021 26279190 PMC4553238

[B6] Chen F. Bai J. Li W. Mei P. Liu H. Li L. (2013). RUNX3 suppresses migration, invasion and angiogenesis of human renal cell carcinoma. *PLOS ONE* 8:e56241. 10.1371/journal.pone.0056241 23457532 PMC3572981

[B7] Cowan C. A. Yokoyama N. Bianchi L. M. Henkemeyer M. Fritzsch B. (2000). EphB2 guides axons at the midline and is necessary for normal vestibular function. *Neuron* 26 417–430. 10.1016/S0896-6273(00)81174-5 10839360

[B8] Daniel T. O. Abrahamson D. (2000). Endothelial signal integration in vascular assembly. *Annu. Rev. Physiol.* 62 649–671. 10.1146/annurev.physiol.62.1.649 10845106

[B9] Egawa N. Lok J. Washida K. Arai K. (2017). Mechanisms of axonal damage and repair after central nervous system injury. *Transl. Stroke Res.* 8 14–21. 10.1007/s12975-016-0495-1 27566737 PMC5243173

[B10] Govek E. E. Newey S. E. Van Aelst L. (2005). The role of the Rho GTPases in neuronal development. *Genes Dev.* 19 1–49. 10.1101/gad.1256405 15630019

[B11] Gu X. S. Ding F. Williams D. F. (2014). Neural tissue engineering options for peripheral nerve regeneration. *Biomaterials* 35 6143–6156. 10.1016/j.biomaterials.2014.04.064 24818883

[B12] Hallock P. T. Xu C. F. Park T. J. Neubert T. A. Curran T. Burden S. J. (2010). Dok-7 regulates neuromuscular synapse formation by recruiting Crk and Crk-L. *Genes Dev.* 24 2451–2461. 10.1101/gad.1977710 21041412 PMC2964755

[B13] Hu Y. W. Jiang J. J. Yan-Gao Wang R. Y. Tu G. J. (2016). MicroRNA-210 promotes sensory axon regeneration of adult mice in vivo and in vitro. *Neurosci. Lett.* 622 61–66. 10.1016/j.neulet.2016.04.034 27102143

[B14] Inoue K. Ozaki S. Shiga T. Ito K. Masuda T. Okado N. (2002). Runx3 controls the axonal projection of proprioceptive dorsal root ganglion neurons. *Nat. Neurosci.* 5 946–954. 10.1038/nn925 12352981

[B15] Jain R. K. Au P. Tam J. Duda D. G. Fukumura D. (2005). Engineering vascularized tissue. *Nat. Biotechnol.* 23 821–823. 10.1038/nbt0705-821 16003365

[B16] Jessen K. R. Mirsky R. (2016). The repair Schwann cell and its function in regenerating nerves. *J. Physiol.* 594 3521–3531. 10.1113/JP270874 26864683 PMC4929314

[B17] Li B. Wang H. Zhou G. Zhang J. Su X. L. Huang Z. F. (2017). VEGF-loaded biomimetic scaffolds: a promising approach to improve angiogenesis and osteogenesis in an ischemic environment. *RSC Adv.* 7 4253–4259. 10.1039/c6ra25294j

[B18] Li S. Y. Liu Q. Q. Wang Y. J. Gu Y. Liu D. Wang C. M. (2013). Differential gene expression profiling and biological process analysis in proximal nerve segments after sciatic nerve transection. *PLOS ONE* 8:e57000. 10.1371/journal.pone.0057000 23437294 PMC3578805

[B19] Lindholm T. Risling M. Carlstedt T. Hammarberg H. Wallquist W. Cullheim S. (2017). Expression of semaphorins, neuropilins, VEGF, and tenascins in rat and human primary sensory neurons after a dorsal root injury. *Front. Neurol.* 8:49. 10.3389/Fneur.2017.00049 28270793 PMC5318460

[B20] Liu X. Hawkes E. Ishimaru T. Tran T. Sretavan D. W. (2006). EphB3: an endogenous mediator of adult axonal plasticity and regrowth after CNS injury. *J. Neurosci.* 26 3087–3101. 10.1523/JNEUROSCI.4797-05.2006 16554460 PMC6674090

[B21] Ljuca F. Drevensek G. (2010). Endothelin-1 induced vascular smooth muscle cell proliferation is mediated by cytochrome P-450 arachidonic acid metabolites. *Bosn. J. Basic Med. Sci.* 10 223–226. 20846129 10.17305/bjbms.2010.2691 PMC5504499

[B22] Makarevich P. I. Boldyreva M. A. Gluhanyuk E. V. Efimenko A. Y. Dergilev K. V. Shevchenko E. K. (2015). Enhanced angiogenesis in ischemic skeletal muscle after transplantation of cell sheets from baculovirus-transduced adipose-derived stromal cells expressing VEGF165. *Stem Cell Res. Ther.* 6 204. 10.1186/S13287-015-0199-6 26503601 PMC4620646

[B23] Mingueneau M. Kreslavsky T. Gray D. Heng T. Cruse R. Ericson J. (2013). The transcriptional landscape of alpha beta T cell differentiation. *Nat. Immunol.* 14 619–632. 10.1038/ni.2590 23644507 PMC3660436

[B24] Novosel E. C. Kleinhans C. Kluger P. J. (2011). Vascularization is the key challenge in tissue engineering. *Adv. Drug Deliv. Rev.* 63 300–311. 10.1016/j.addr.2011.03.004 21396416

[B25] Ou M. Dang Y. Mazzuca M. Q. Basile R. Khalil R. A. (2014). Adaptive regulation of endothelin receptor type-A and type-B in vascular smooth muscle cells during pregnancy in rats. *J. Cell. Physiol.* 229 489–501. 10.1002/jcp.24469 24105843 PMC3867575

[B26] Park I. Lee H. S. (2015). EphB/ephrinB signaling in cell adhesion and migration. *Mol. Cells* 38 14–19. 10.14348/molcells.2015.2116 25475547 PMC4314128

[B27] Potente M. Gerhardt H. Carmeliet P. (2011). Basic and therapeutic aspects of angiogenesis. *Cell* 146 873–887. 10.1016/j.cell.2011.08.039 21925313

[B28] Qian Y. Liu K. J. Chen Y. Flynn D. C. Castranova V. Shi X. (2004). Cdc42 regulates arsenic-induced NADPH oxidase activation and cell migration through actin filament reorganization. *J. Biol. Chem.* 280 3875–3884. 10.1074/jbc.M403788200 15492012

[B29] Rouwkema J. Khademhosseini A. (2016). Vascularization and angiogenesis in tissue engineering: beyond creating static networks. *Trends Biotechnol.* 34 733–745. 10.1016/j.tibtech.2016.03.002 27032730

[B30] Salani D. Taraboletti G. Rosano L. Di Castro V. Borsotti P. Giavazzi R. (2000). Endothelin-1 induces an angiogenic phenotype in cultured endothelial cells and stimulates neovascularization in vivo. *Am. J. Pathol.* 157 1703–1711. 10.1016/S0002-9440(10)64807-9 11073829 PMC1885730

[B31] Schulz J. Franke K. Frick M. Schumacher S. (2016). Different roles of the small GTPases Rac1, Cdc42, and RhoG in CALEB/NGC-induced dendritic tree complexity. *J. Neurochem.* 139 26–39. 10.1111/jnc.13735 27412363

[B32] Song K. Wu H. Rahman H. N. A. Dong Y. Z. Wen A. Y. Brophy M. L. (2017). Endothelial epsins as regulators and potential therapeutic targets of tumor angiogenesis. *Cell. Mol. Life Sci.* 74 393–398. 10.1007/s00018-016-2347-2 27572288 PMC5243150

[B33] Stenberg L. Kodama A. Lindwall-Blom C. Dahlin L. B. (2016). Nerve regeneration in chitosan conduits and in autologous nerve grafts in healthy and in type2 diabetic Goto-Kakizaki rats. *Eur. J. Neurosci.* 43 463–473. 10.1111/ejn.13068 26355640

[B34] Tezuka T. Inoue A. Hoshi T. Weatherbee S. D. Burgess R. W. Ueta R. (2014). The MuSK activator agrin has a separate role essential for postnatal maintenance of neuromuscular synapses. *Proc. Natl. Acad. Sci. U.S.A.* 111 16556–16561. 10.1073/pnas.1408409111 25368159 PMC4246289

[B35] Wang H. K. Wang Y. X. Xue C. B. Li Z. M. Y. Huang J. Zhao Y. H. (2016). Angiogenesis in tissue-engineered nerves evaluated objectively using MICROFIL perfusion and micro-CT scanning. *Neural Regen. Res.* 11 168–173. 10.4103/1673-5374.175065 26981108 PMC4774213

[B36] Webber C. Zochodne D. (2010). The nerve regenerative microenvironment. *Exp. Neurol.* 223 51–59. 10.1016/j.expneurol.2009.05.037 19501085

[B37] Wild R. Klems A. Takamiya M. Hayashi Y. Strahle U. Ando K. (2017). Neuronal sFlt1 and Vegfaa determine venous sprouting and spinal cord vascularization. *Nat. Commun.* 8:13991. 10.1038/Ncomms13991 28071661 PMC5234075

[B38] Yahata Y. Shirakata Y. Tokumaru S. Yamasaki K. Sayama K. Hanakawa Y. (2003). Nuclear translocation of phosphorylated STAT3 is essential for vascular endothelial growth factor-induced human dermal microvascular endothelial cell migration and tube formation. *J. Biol. Chem.* 278 40026–40031. 10.1074/jbc.M301866200 12874294

[B39] Yamao M. Naoki H. Kunida K. Aoki K. Matsuda M. Ishii S. (2015). Distinct predictive performance of Rac1 and Cdc42 in cell migration. *Sci. Rep.* 5:17527. 10.1038/Srep17527 26634649 PMC4669460

[B40] Yamauchi J. Chan J. R. Shooter E. M. (2003). Neurotrophin 3 activation of TrkC induces Schwann cell migration through the c-Jun N-terminal kinase pathway. *Proc. Natl. Acad. Sci. U.S.A.* 100 14421–14426. 10.1073/pnas.2336152100 14614136 PMC283607

