# Supplementary figures and images for: Overlapping Mechanisms of Peripheral Nerve Regeneration and Angiogenesis Following Sciatic Nerve Transection
Source: Front Cell Neurosci. 2017 Oct 11;11:323. doi: 10.3389/fncel.2017.00323 (PMC5649188; doi:10.3389/fncel.2017.00323)

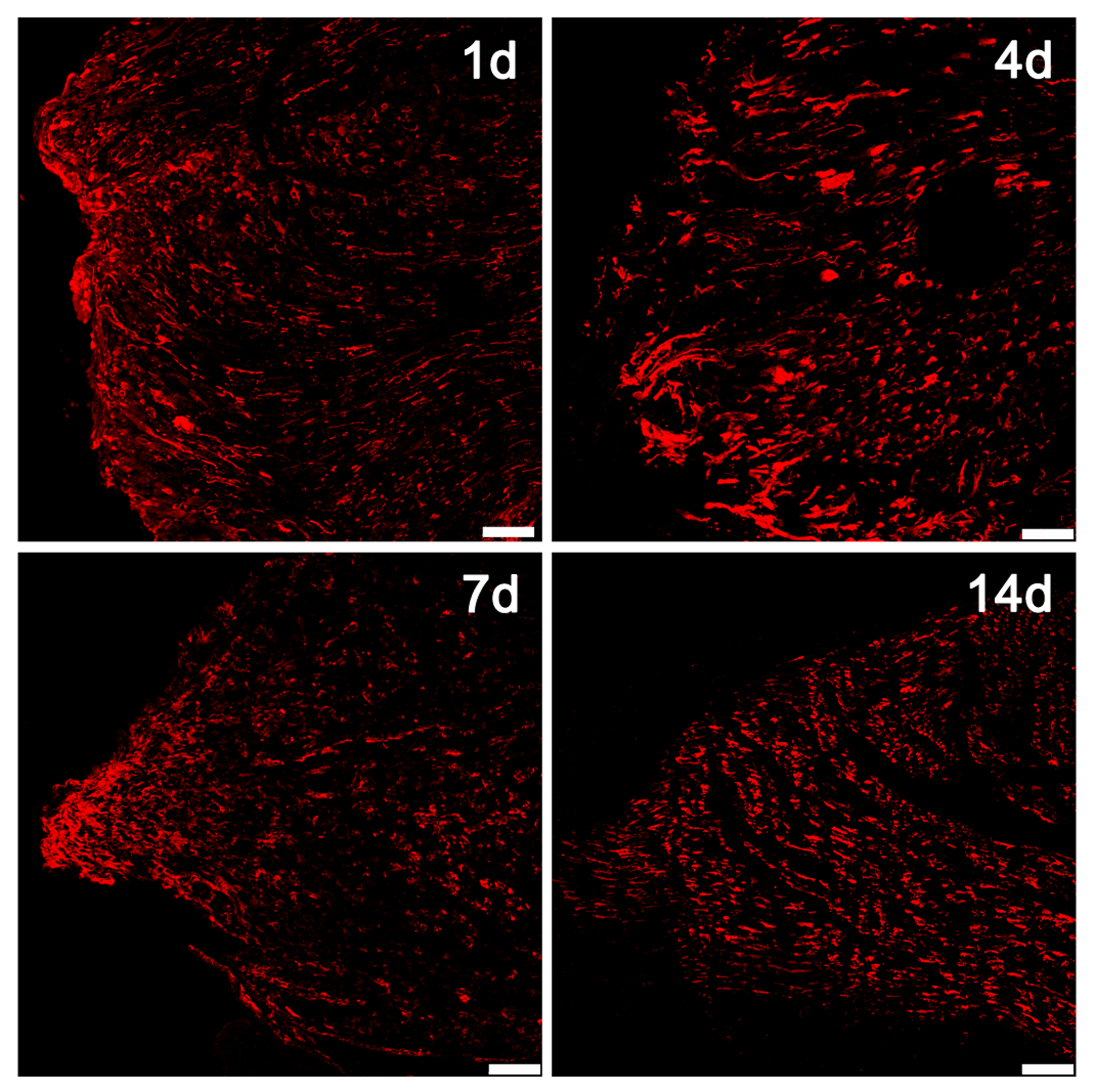

Supplement: FIGURE S1 — Axonal regeneration of proximal nerve stump post sciatic nerve transection. Immunofluorescent staining of anti-NF-200 (red) at 1, 4, 7, and 14 days post nerve transection of longitudinal sections, respectively. The regenerated axons extended to the distal stump gradually over time. The forefront of regenerated axons growing from proximal nerve stump could be seen obviously at 7 and 14 days after nerve transection. Scale bar, 75 μm. [file Image_1.TIF]
